# Peripheral Blood miRome Identified miR-155 as Potential Biomarker of MetS and Cardiometabolic Risk in Obese Patients

**DOI:** 10.3390/ijms22031468

**Published:** 2021-02-02

**Authors:** Alvaro Cerda, Adonai Aralim Amaral, Raquel de Oliveira, Tamiris Invencioni Moraes, Aécio Assunção Braga, Magda Elizabeth Graciano-Saldarriaga, Cristina Moreno Fajardo, Thiago Dominguez Crespo Hirata, Vivian Bonezi, Antony Brayan Campos-Salazar, Egidio Lima Dorea, Marcia Martins Silveira Bernik, Mario Hiroyuki Hirata, Rosario Dominguez Crespo Hirata

**Affiliations:** 1 Center of Excellence in Translational Medicine, CEMT-BIOREN & Department of Basic Sciences, Universidad de La Frontera, Av. Alemania 0458, Temuco 4810296, Chile; 2 Department of Clinical and Toxicological Analyses, School of Pharmaceutical Sciences, University of Sao Paulo, Av. Prof. Lineu Prestes 580, Sao Paulo 05508-000, Brazil; adonai.amaral@gmail.com (A.A.A.); quel_sheva@yahoo.com.br (R.d.O.); ta3003@hotmail.com (T.I.M.); aeciobraga86@gmail.com (A.A.B.); melizabethgs04@gmail.com (M.E.G.-S.); cris.mf01@hotmail.com (C.M.F.); thiagodch@gmail.com (T.D.C.H.); vivian.bonezi@gmail.com (V.B.); antonybcampos@gmail.com (A.B.C.-S.); mhhirata@usp.br (M.H.H.); rosariohirata@usp.br (R.D.C.H.); 3 University Hospital, University of Sao Paulo, Av. Prof. Lineu Prestes 2565, Sao Paulo 05508-000, Brazil; egidiodr@gmail.com (E.L.D.); marcia.bernik@gmail.com (M.M.S.B.)

**Keywords:** obesity, metabolic syndrome, microRNAs, miR-155, *CEBPB*, cardiometabolic risk

## Abstract

This study explored circulating miRNAs and target genes associated with metabolic syndrome (MetS) and cardiometabolic risk in obese patients. Small-RNA sequencing was used to assess the peripheral blood miRNome of 12 obese subjects (6 MetS and 6 non-MetS). Differentially expressed miRNAs and target genes were further analyzed by qPCR in a larger sample of obese patients (48 MetS and 32 non-MetS). miRNA:mRNA interactions were studied using in silico tools. miRNome analysis identified 10 downregulated miRNAs in MetS compared to non-Met patients (*p* < 0.05). In silico studies revealed three miRNAs (miR-155, miR-181a, and let-7a) and their predictive targets (CCAAT/enhancer-binding protein beta—*CEBPB*, KRAS proto-oncogene, GTPase—*KRAS* and suppressor of cytokine signaling 1—*SOCS1*) with a potential role in the insulin receptor signaling pathway. miR-155 expression was reduced and *CEBPB* mRNA levels were increased in MetS patients (*p* < 0.05), and these effects were correlated with the number of MetS diagnostic criteria (*p* < 0.05). Increased HOMA-IR (>7.6) was associated with low miR-155 levels, high *CEBPB* expression, and serum hsCRP (*p* < 0.05). miR-155 was negatively correlated with *CEBPB*, HOMA-IR, and plasma fibrinogen, and positively correlated with serum adiponectin (*p* < 0.05). Downregulation of circulating miR-155 is associated with insulin resistance, poor glycemic control, and increased MetS-related cardiometabolic risk, and these effects are potentially mediated by interaction with *CEBPB.*

## 1. Introduction

Metabolic syndrome (MetS) is a clinical condition of excessive body adiposity and several metabolic alterations, such as insulin resistance, impaired glucose tolerance, dyslipidemia, hypertension, and obesity, which have been shown to increase the risk of cardiovascular diseases [1]. The excessive adiposity stimulates the production of pro-inflammatory cytokines, which leads to a chronic inflammatory status that ultimately results in insulin resistance and other metabolic alterations [2].

Genetic factors have been implicated in the pathophysiology of obesity and MetS. Genome-wide and candidate genes association studies have shown the role of variants in genes of the leptin-melanocortin pathway on body mass index (BMI), adiposity, and metabolic dysregulation related to obesity and MetS in several populations [3,4,5]. Variants in genes involved in the insulin signaling pathway have also been associated with insulin resistance intrinsically related to MetS [6]. Our previous studies demonstrated that variants in *LEP* and *LEPR* are associated with adiposity and metabolic alterations and that *ADIPOQ* and *IL6* variants contribute to cardiometabolic risk in Brazilian obese subjects [7,8]. We also recently reported that polymorphisms at *LEP, LEPR,* and *MC4R* may be useful markers of obesity-related cardiometabolic alterations in a cohort from South Chile [9].

Environmental and epigenetic factors have also been involved in the pathogenesis of obesity and obesity-related complications [4]. DNA methylation, histone modifications, and non-coding RNAs, such as microRNAs (miRNAs), are involved in epigenetic mechanisms that regulate gene expression and biological processes. miRNAs have been shown to modulate adipogenesis, insulin signaling, and other metabolic pathways, and their dysregulation was associated with obesity and MetS [10,11,12].

Circulating miRNAs have been proposed as potential biomarkers for diagnosis, prognosis, and follow-up of metabolic diseases [13,14,15]. In this way, large-scale approaches, such as microarray and next-generation sequencing, have accelerated the search for coding and non-coding RNAs to elucidate the pathophysiological mechanisms involved in cardiometabolic diseases [16,17].

This study was designed to analyze the peripheral blood miRNome using a small RNA-sequencing strategy to identify miRNAs and target genes that are associated with MetS and cardiometabolic risk in obese patients.

## 2. Subjects and Methods

### 2.1. Patients and Study Design

A group of 80 obese patients were recruited at the University Hospital of the University of Sao Paulo (HU/USP) and at the Institute Dante Pazzanese of Cardiology (IDPC), Sao Paulo, Brazil. Subjects with thyroid disease or other endocrinopathies, hepatic, and renal diseases, and any secondary form of obesity were not included in the study.

Anthropometric measurements, such as BMI, waist circumference, waist-to-hip ratio, and body fat mass measured by bioimpedance, were obtained for each participant. Obesity was defined according to the World Health Organization as BMI ≥30 Kg/m^2^. Clinical and demographic data such as gender, tobacco smoking, alcohol consumption, dyslipidemia, hypertension, and type 2 diabetes (T2D) were recorded. Patients were grouped as having or not MetS, according to the International Diabetes Federation criteria [18].

**Figure 1:**
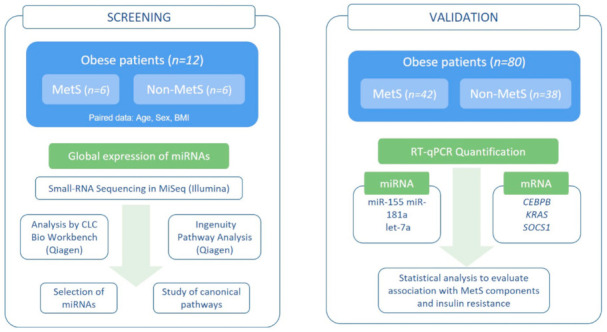
Workflow of the study protocol. MetS: Metabolic Syndrome; BMI: Body mass index.

The study protocol was performed using a two-stage strategy (Figure 1). In the first stage (Screening study), the global expression of miRNAs (miRNome) was analyzed by small-RNA sequencing, in peripheral blood of six MetS and six non-MetS obese patients. In the second stage (Validation study), the differentially expressed miRNAs and the selected target genes were analyzed by reverse transcription quantitative PCR (RT-qPCR) in peripheral blood of the whole cohort (42 MetS and 38 non-MetS).

The study protocol was approved by the Ethics Committees of the HU/USP (Protocol # 812/08, approved in 16 May 2008), School of Pharmaceutical Sciences of University of Sao Paulo (Protocol # 471, approved in 26 May 2008), and IDPC (Protocol # 4134, approved in 7 October 2014). All subjects were informed about the study protocol and agreed to participate as volunteers by signing the informed consent form.

### 2.2. Clinical Laboratory Analyses

Blood samples were collected after an overnight (12 h) fast. Serum glucose, total cholesterol, high-density lipoprotein (HDL) cholesterol, and triglycerides were measured by standard enzymatic colorimetric methods. Low-density lipoprotein (LDL) and very low-density (VLDL) cholesterol were estimated by Friedewald’s formula. Glycated hemoglobin (HbA1c) was measured in EDTA-anticoagulated blood by affinity chromatography using the D10 Hemoglobin Testing System (Biorad^®^, San Francisco, CA, USA). Insulin was determined by chemiluminescence (Siemens Healthcare Diagnosis Inc., Tarrytown, NY, USA) and the homeostasis model assessment of insulin resistance (HOMA-IR) was calculated. Fibrinogen, and high-sensitivity C-reactive protein (hsCRP) were analyzed by laboratory standard assays. Interleukin 1β (IL-1β), interleukin 6 (IL-6), adiponectin, leptin and resistin were measured using the Luminex^®^ 100TM detection system (Luminex Corporation, Austin, TX, USA).

### 2.3. RNA Extraction and Analysis

Peripheral blood samples were collected in Tempus™ Blood for RNA stabilization and stored at −20 °C until RNA extraction. Total RNA, including miRNA fraction, was extracted from stabilized Tempus™ Blood tubes using the MagMAX™ for Stabilized Blood Tubes RNA Isolation Kit (Applied Biosystems, Foster City, CA, USA) following the manufacturer’s suggested protocol. RNA concentration and purity were measured by spectrophotometry using NanoDrop^®^ (NanoDrop Technologies INC., DE, USA). RNA integrity was evaluated in the Bioanalyzer^®^2100 using the Agilent RNA 6000 NanoChip Kit (Agilent Technologies Inc., Santa Clara, CA, USA). Samples with RNA integrity number (RIN) lower than 8 were not used for small RNA sequencing experiments, whereas a RIN >6 was considered for miRNA and mRNA expression analyses.

### 2.4. miRome Analysis by RNA-Seq

The miRome was analyzed by small-RNA sequencing. Briefly, Illumina TruSeq Small RNA Sample Preparation Kit (Illumina, Inc., San Diego, CA, USA) was used to prepare libraries of miRNAs using 1 µg total RNA. Libraries of six combined samples (fragment size 147 bp) were isolated through 6% polyacrylamide gel electrophoresis and then validated using the High sensitivity DNA chip (Agilent Technologies, Santa Clara, CA, USA) and the Bioanalyzer 2100 (Agilent Technologies Inc., Santa Clara, CA, USA). Purified libraries were quantified using the Kapa library quantification kit (Kapabiosystems, Boston, MA, USA) and the 7500 Fast Real-Time PCR system (Applied Biosystem, Forest City, IA, USA). Libraries at 2 mM were denaturated with 1:1 NaOH 1N by 5 min, diluted at 15 pM and then sequenced using the MiSeq^®^ Reagent Kit (50 cycles) and the Illumina MiSeq (Illumina, Inc., San Diego, CA, USA).

Demultiplexing and the quality of raw sequence reads was initially checked using the MiSeq Reporter software v3.1 (Illumina, Inc., San Diego, CA, USA). Only reads with Q-score >30 were included in further analysis. The CLC Bio Workbench software (Qiagen, Hilden, Germany) was used for complementary quality control, sequence annotation, and to obtain read counts. miRNA sequenced reads were mapped against the miRbase v22.1 and grouped according mature sequence of miRNAs. Comparative analysis was performed by the Baggerly test and correction by false discovery rate (FDR) for multiple tests using the CLC Bio Workbench software.

### 2.5. In Silico Analysis for Selection of miRNAs Target Genes

miRNAs differentially expressed in the screening study were used to select target genes using the Ingenuity Pathway Analysis (IPA) software (Qiagen, Redwood City, CA, USA). Potential canonical pathways containing the selected genes were found by the Pathway Analysis tool. The miRNA:mRNA interactions from these pathways were filtered with the miRNA Target filter tool to keep interactions with experimental evidence or with a high level of prediction. The remaining interactions were considered related to obesity and its metabolic alterations.

### 2.6. Expression of Selected miRNAs by Stem Loop RT-qPCR

In the stage 2 (Validation study), the expression of the selected miRNAs (hsa-miR-155-5p, MIMAT0000646; hsa-miR-181a, MIMAT0000256; and hsa-let-7a-5p, MIMAT 0000062) was measured by stem loop TaqMan RT-qPCR (Life Technologies, Carlsbad, CA, USA). Specific stem loop primers were used to synthetize the cDNA from 10 ng total RNA using the micro-RNA transcription kit (Life Technologies, CA, USA). RT-qPCR assays were carried out using predesigned assays (miR-155, ID: 002623; miR-181a, ID: 00048; and let-7a, ID: 000377) (Life Technologies CA, USA). RNU24 was selected as the most stable endogenous reference among 4 small nucleolar RNAs (RNU24, RNU6B, RNU58, RNU44), which were evaluated by using the GeNorm software [19]. All samples were assayed in duplicate, and the relative miRNA expression was analyzed using the comparative C_T_ method using the formula 2^−ΔCt^ [20].

### 2.7. Expression of Target mRNAs by qPCR

The mRNA expression of selected genes (CCAAT/enhancer-binding protein beta—*CEBPB,* KRAS proto-oncogene, GTPase—*KRAS,* and suppressor of cytokine signaling 1—*SOCS1*) was measured by TaqMan qPCR. Briefly, cDNA was produced from 1 μg of total RNA, extracted from peripheral blood, using the Superscript™ II Reverse Transcriptase (Invitrogen-Life Technologies, CA, USA). The qPCR assays were carried out in 96 well plates using the predesigned TaqMan assays (*CEBPB*, Hs00270923_s1; *KRAS*, Hs00364284_g1; and *SOCS1*, Hs00705164_s1) (Life Technologies, CA, USA) and the 7500 Fast Real-Time PCR system (Applied Biosystem, Forest City, IA, USA).

Six reference genes (*UBC*, *GAPD*, *B2M*, *HPRTI*, *SDHA* and *HMBS*) were tested and analyzed using the GeNorm software [http://medgen.ugent.be/genorm]. The most stable gene in the experimental conditions was *B2M*. The sequence of primers and probe used for *B2M* are described as follows: forward, 5′-TGCTGTCTCCATGTTTGATGTATCT-3′; reverse, 5′-TCTCTGCTCCCCACCTCTAAGT-3′; and probe, 5′Vic-CTCCACAGGTAGCTCT- MGB/NFQ 3′. All cDNA samples were assayed in duplicate and the relative quantification of gene expression was analyzed using the comparative Ct method using the formula 2^−ΔCt^ [20].

### 2.8. Statistical Analysis

Statistical analyses were performed using SPSS v.15 (SPSS Inc., IBM, Chicago, IL, USA) and Prism v.5.0 (Graph Pad Software Inc., La Jolla, CA, USA). Categorical variables were compared by chi-square test. The distribution of continuous variables was analyzed using the Kolmogorov-Smirnov test. Variables with normal distribution were compared by *t*-test, and those with skewed distributions were compared using the Mann–Whitney U test or Kruskal-Wallis test and Dunn’s post-hoc test. The correlation between continuous variables was explored using the Spearman correlation test. Statistical significance was set for *p*-value < 0.05.

## 3. Results

### 3.1. Characteristics of the Study Groups

The main characteristics of the total group obese patients grouped according to the MetS status are shown in the Table 1. As expected, MetS group has higher frequency of hypertension, dyslipidemia and insulin resistance, and higher values of BMI, waist circumference and waist-hip ratio (*p* < 0.05). In addition, the altered metabolic profile exposes the MetS group to increased cardiometabolic risk.

Characteristics of the obese patients selected for the screening study (*n* = 12) also showed worse glycemic control (hyperglycemia, insulinemia, HOMA-IR, and HbA1c) in MetS than in non-MetS patients (*p* < 0.05) (Table S1). Altered anthropometric and serum lipid variables were also observed in MetS of this sample but without statistical significance.

### 3.2. miRNome in Peripheral Blood (Screening Study)

Small-RNA sequencing data resulted in approximately 21.3 million (M) raw sequencing reads, with a mean value of 1.8 reads per sample ranging from 1.2 M to 2.2 million (M) reads per sample, which could occur due to individual (MetS and non-MetS patients) and experimental (RNA extraction, library preparation) variability. After the adapter and quality trimming step, and filtering by quality (Q-score > 30) we retained approximatelly 90% (19.1M) of the initial sequencing reads, which shows the high quality of the dataset. Filtered reads ranging 1.1 to 1.9 M reads per sample were mapped to 812 unique known miRNA sequences from similar proportions of mature miRNAs and miRNA precursors using the miRBase v22.1. The hsa-miR-486-5p miRNA was the most abundant in all samples studied, representing approximately 80% of the total identified miRNAs.

**Figure 2:**
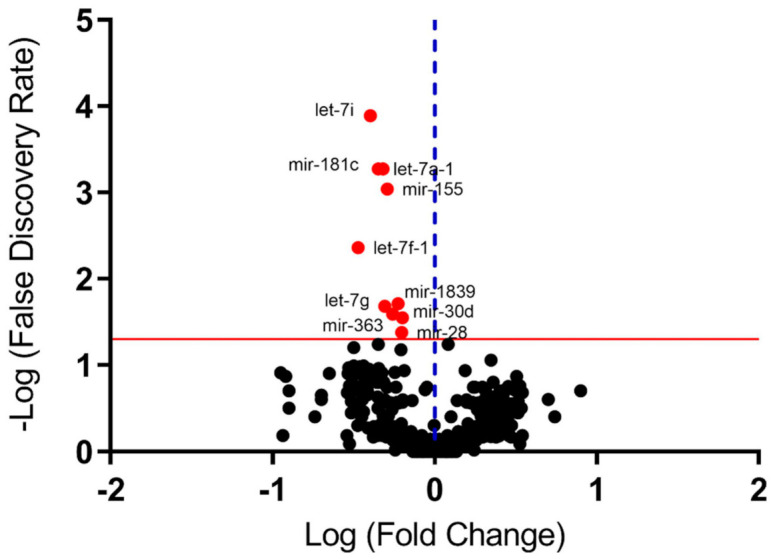
Volcano plot representing differentially expressed miRNAs at the screening study in peripheral blood of MetS and non-MetS obese subjects. Fold change represents the differential expression of miRs between MetS (*n* = 6) and non-MetS (*n* = 6) groups. Comparative analysis was performed by the Baggerley test corrected by false discovery rate (FDR) for multiple tests using the CLC Genomic Workbench software (Qiagen, Hilden, Germany). The red line indicates FDR = 0.05 and the dots above this line have FDR < 0.05. The dotted blue line indicates no change in expression values. Red dots represent miRNAs downregulated in MetS obese group.

The results of the miRNAs global expression in peripheral blood showed 10 miRNAs differentially expressed in MetS (*n* = 6) compared to non-MetS (*n* = 6) obese patients, after correction for multiple tests (Figure 2; Table 2; Table S2). The miRNAs from the let family (let-7a-1, let-7f-1, let-7g, let-7i) and other miRNAs (miR-28, miR-30d, miR-155, miR-181a, miR-363 and miR-1839) were downregulated in MetS patients.

### 3.3. Selected miR:mRNA Interactions Study

The interactions between the differentially expressed miRNAs and target genes were studied using the IPA canonical pathway. Twelve genes were selected, and at a level of experimental evidence or with a high level of prediction, the insulin receptor signaling pathway was the main canonical pathway (Table S3). Further analysis using the IPA miRNA Target Filter tool showed three miRNAs (miR-155, miR-181a and let-7a) as candidates to have relevant biological role by targeting genes involved in the regulation of the insulin receptor signaling pathway, such as *CEBPB*, *KRAS,* and *SOCS1* (Figure S1).

### 3.4. Expression of Selected miRNAs by qPCR (Validation Study)

**Figure 3:**
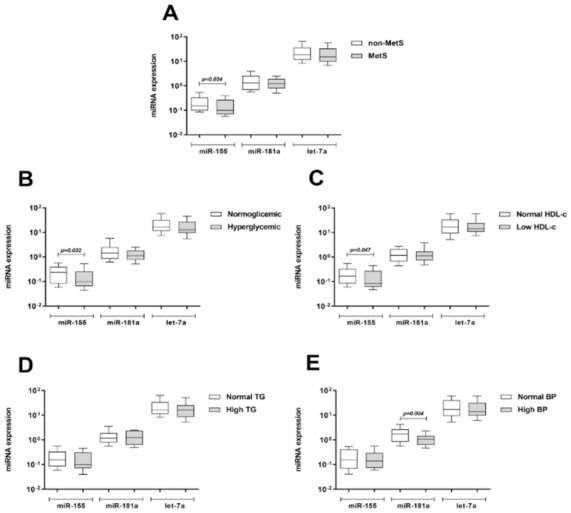
Expression of miR-155, miR-181a, and let-7a in peripheral blood of obese patients (*n* = 80) (Validation study). Patients were grouped according to the MetS status (**A**) and according to the IDF criteria for MetS diagnosis: (**B**) Hyperglycemia; (**C**) low HDL-c; (**D**) high triglycerides; and (**E**) high blood pressure. miRNA expression was measured using stem-loop Taqman RT-qPCR. Data are shown as box plots and were compared by Mann–Whitney U test. MetS: Metabolic syndrome; HDL-c: High-density lipoprotein cholesterol; TG: Triglycerides; BP: Blood pressure.

The differential expression of the miR-155, miR-181a and let-7a was further analyzed in peripheral blood the total group of obese patients (*n* = 80), however, only miR-155 remained downregulated in MetS patients compared to non-MetS (*p* = 0.034, Figure 3A). The expression of these miRNAs was also analyzed in obese patients with different criteria for MetS diagnosis (Figure 3B–E). Expression of miR-155 was lower in patients with hyperglycemia (*p* = 0.032, Figure 3B) and low HDL-c (*p* = 0.047, Figure 3C). miR-181a expression was lower in hypertensive patients (*p* = 0.004, Figure 3E), whereas no difference was observed for let-7a expression according any individual MetS criterion.

### 3.5. Expression of Target Genes in Peripheral Blood

**Figure 4:**
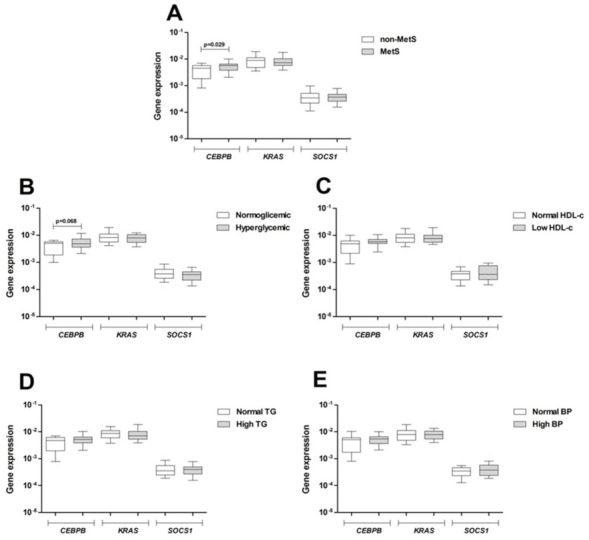
*CEBPB*, *KRAS* and *SOCS1* mRNA expression of in peripheral blood of obese patients (*n* = 80). Patients were grouped according to the MetS status (**A**) and according to the IDF criteria for MetS diagnosis: (**B**) Hyperglycemia; (**C**) low HDL-c; (**D**) high TG; and (**E**) high BP. Data are shown as box plots and were compared by Mann–Whitney U test. MetS: Metabolic syndrome; BP: Blood pressure; HDL-c: High-density lipoprotein cholesterol; TG: Triglycerides.

Expression of *CEBPB*, *KRAS,* and *SOCS1* was also evaluated in peripheral blood of the total cohort of obese patients, as shown in Figure 4. *CEBPB* mRNA was higher in MetS than in non-MetS patients (*p* = 0.029, Figure 4A), and also a trend to higher values was observed in hyperglycemic compared with normoglycemic patients (*p* = 0.068) (Figure 4B). On the other hand, expression of *KRAS* and *SOCS1* were not influenced by MetS diagnosis criteria.

### 3.6. Influence of MetS Criteria and Insulin Resistance on miRNA and mRNA Expression

We further explored the influence of MetS criteria on miRNA expression and a markedly reduction of the miR-155 expression was found in obese patients with 3 or 4 MetS criteria, which is indicative of high cardiometabolic risk (*p* < 0.05) (Figure S2). Moreover, a progressive increase in *CEBPB* mRNA expression was observed as obese patients meet a greater number of additional criteria of MetS (Figure S2).

**Figure 5:**
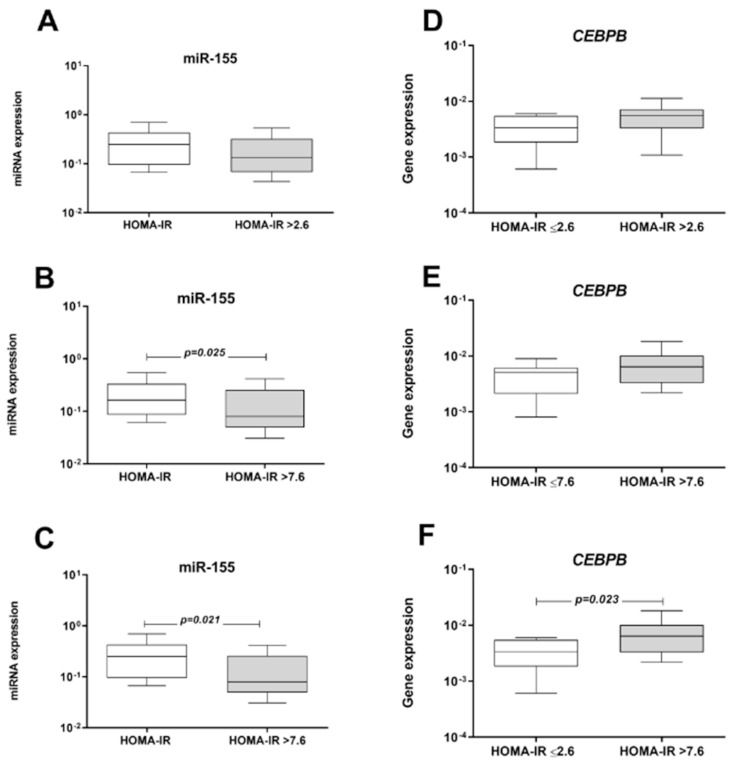
Expression of miR-155 and *CEBPB* mRNA according to HOMA-IR values of obese patients (n = 80). Data are shown as box plots and were compared by the Mann–Whitney U test. *p* < 0.05. Patients were grouped according to the HOMA-IR values using the first quartile (25th percentile-p25, HOMA-IR = 2.6) and the third quartile (75th percentile-p75, HOMA-IR = 7.6) as cut-off values. HOMA-IR: Homeostasis model assessment of insulin resistance.

In order to investigate the influence of insulin resistance on miRNA and mRNA expression, HOMA-IR of the obese patients were grouped in quartiles, and the first quartile (25th percentile-p25, HOMA-IR < 2.6) and the third quartile (75th percentile-p75, HOMA-IR > 7.6) were used as cut-off values (Figure 5). miR-155 expression was lower patients with HOMA-IR >7.6 in comparison with HOMA-IR ≤ 7.6 (Figure 5B, *p* < 0.05) and ≤2.6 (*p* < 0.05, Figure 5C). Obese patients with HOMA-IR > 7.6 had higher *CEBPB* mRNA expression than patients with HOMA-IR ≤ 2.6 (*p* < 0.05, Figure 5F). The correlation analysis also showed that miR-155 expression was inversely correlated with *CEBPB* mRNA levels in peripheral blood, HOMA-IR values and fibrinogen concentrations in obese patients (*p* < 0.05, Table 3). On the other hand, positive correlation was found between miR-155 expression and adiponectin concentration (*p* = 0.017, Table 3).

The influence of insulin resistance according to HOMA-IR cut-off values on inflammatory biomarkers and adipokines of obese subjects was also analyzed (Table S4), and we found no differences for HOMA-IR > 2.6 (p25). Interestingly, obese subjects with HOMA-IR > 7.6 (p75) had higher concentrations of fibrinogen and hsCRP, and lower adiponectin levels in comparison with those with HOMA-IR ≤ 7.6 (*p* < 0.05). These results indicate that subjects with a p75 cut-off were at increased cardiometabolic risk.

## 4. Discussion

This study identified the association of miRNAs expression in peripheral blood with cardiometabolic risk in obese individuals, mainly related to the development of insulin resistance.

The miRNome analysis (Screening study) combined with in silico tools selected 10 miRNAs, of which three (miR-155, miR-181a, and let-7a) and their targets (*CEBPB*, *KRAS,* and *SOCS1*) were predicted to be involved in the insulin receptor signaling pathway, as previously reported [21]. These selected miRNAs were further analyzed in a larger cohort of obese patients by qPCR (Validation study), and only miR-155 remained downregulated in obese patients with MetS, hyperglycemia, and insulin resistance. Analysis of the target genes revealed that *CEBPB*, but not *KRAS,* and *SOCS1* was upregulated in obese patients MetS and insulin resistance.

miR-155 is encoded by the MIR155 host gene (*MIR155HG*) and plays a critical role in various physiological and pathological processes, such as hematopoiesis, immune response, inflammation, neoplasia, and cardiovascular and metabolic diseases [21,22,23]. miR-155 also participates in adipogenesis by regulating the expression of *CEBPB* and *CREB1* (cAMP response element-binding protein 1), early adipogenic transcription factors [24]. miR-155 and other miRNAs were suggested to be involved in adipose tissue dysfunction and the development of obesity-associated disorders, including insulin resistance [25].

Blood-based biomarkers have been explored to evaluate the risk of MetS and other cardiometabolic conditions associated with MetS [15]. This approach included the investigation of differential expression of miRNAs in blood cells [26,27], extracellular vesicles [28], and plasma or serum [15,29,30,31,32,33,34]. This particular interest in miRNAs as markers of metabolic diseases has emerged from the fact that they appear to be causally and mechanistically involved in events that contribute to MetS [15].

In this cohort, MetS patients had decreased miR-155 levels in peripheral blood, particularly related to hyperglycemia and insulin resistance. These findings are consistent with previous studies describing miR-155 as being downregulated in different tissues and experimental models related to impaired glucose metabolism and T2D [35]. Klöting et al. reported miR-155 downregulation in subcutaneous adipose tissue of T2D patients as compared with subjects with normal glucose tolerance [36]. In a rat diabetic model, miR-155 was also markedly reduced in peripheral blood mononuclear cells (PBMC) and other tissues compared to non-diabetic rats [37].

Reduced levels of miR-155 were also reported in PBMC of T2D patients compared to control subjects, and the miR-155 expression was directly correlated with HbA1c, glucose, and BMI [38]. miR-155 was also found downregulated in serum from T2D patients compared to healthy controls [39]. Downregulation of miR-155 expression was observed in untreated and LPS-treated PBMC of diabetic patients in relation to healthy controls [40]. miR-155 expression was also markedly decreased in the livers and peripheral blood of patients with non-alcoholic fatty liver disease, which is a common complication of obesity [41]. Moreover, the expression of circulating miR-155 was significantly downregulated in coronary artery disease (CAD), being positively correlated to triglycerides and total cholesterol levels in these patients [42]. These works evaluated differentially expressed miRNAs using a study design with extreme phenotypes, T2D, obese, or CAD patients and healthy controls.

An important extension of this study is the miR-155 expression in peripheral blood as a potential biomarker to discriminate obese patients with a high cardiometabolic risk, a question even more necessary due to the consistent increase in the prevalence of obesity worldwide. Reinforcing this hypothesis, the miR-155 levels were progressively reduced as the number of additional criteria for MetS diagnosis increased in this cohort of obese patients.

Regarding the mechanistic insights of the role miR-155 downregulation in glucose homeostasis and insulin sensitivity, some works have proposed different experimental approaches using in vitro and animal models [21]. An enlightening study by Lin et al. provided evidence that transgenic mice overexpressing miR-155 have hypoglycemia, improved glucose tolerance, and insulin sensitivity, whereas miR-155 deficiency caused the opposite effects [39]. It was suggested that miR-155 is a positive regulator of insulin sensitivity by repressing important negative regulators of insulin signaling, such as *CEBPB*, *SOCS1*, and *HDAC4*, which were proven to be direct targets of miR-155. In this way, our work demonstrated that miR-155 expression in peripheral blood was negatively correlated with *CEBPB* expression and with HOMA-IR as well, in obese patients. Moreover, *CEBPB* mRNA was increased in MetS patients, and a tendency to increased values was observed in individuals with hyperglycemia. Similar to our findings, Lin et al. also demonstrated an inverse correlation of serum miR-155 with HOMA-IR [39].

The hyperlipidemia-associated endotoxemia in mice was also shown to induce upregulation of miR-155 in pancreatic β-cells, improving glucose metabolism and adaptation of β-cells to obesity-induced insulin resistance [43]. This effect resulted from miR-155 suppression of *Mafb*, which promoted β-cell function through IL-6-induced GLP-1 production in α-cells. Moreover, reduced GLP-1 levels were associated with increased obesity progression, dyslipidemia, and atherosclerosis in hyperlipidemic miR-155 knockout mice [43].

The predictive analysis showed that miR-155 targets *CEBPB*. A previous work demonstrated by a luciferase assay that *CEBPB* translation is suppressed by miR-155 through interaction with the 3′UTR of *CEBPB* mRNA [44]. In this study, obese patients with insulin resistance (high HOMA-IR) showed reduced miR-155 and increased *CEBPB* expression in peripheral blood. This result confirms the interaction between miR-155 and *CEBPB* in regulating insulin signaling and controlling glycemic control, as aforementioned [39].

We found an inverse correlation of miR-155 expression with HOMA-IR and fibrinogen levels, and a positive correlation with adiponectin, an anti-inflammatory adipokine that protects against obesity-linked metabolic disease [45]. In diabetic patients and rat models, expression of miR-155 in PBMC was also negatively correlated with TNFα, IL-6, and NF-kB activity, which was associated with diabetes complications) [37]. miR-155 was previously shown to regulate inflammatory cytokine production (IL-6, IL-10 and TNFα) in tumor-activated macrophages via targeting *CEBPB* [44].

This study has some limitations, such as the small sample size, which restrains the power of statistical tests. In this way, this pilot study waits for further confirmation in other populations using larger sample sizes of obese individuals at high cardiometabolic risk. In addition, even though a homogeneous group of obese patients was evaluated in this study, the influence of diet on the expression of circulating miR-155 remains unknown, so it would be useful to use a more controlled sample regarding food intake.

## 5. Conclusions

The results from this study are suggestive that a low expression of miR-155 in peripheral blood may be a helpful biomarker to discriminate obese patients at high cardiometabolic risk, mainly regarding insulin resistance and poor glycemic control. Moreover, this effect seems to be mediated, at least in part, by the interaction of miR-155 with *CEBPB*.

## Figures and Tables

**Table 1:** Main characteristic of the obese patients according to the MetS status.

	Variables	Total Group (*n* = 80)	MetS (*n* = 48)	non-MetS (*n* = 32)	*p*-Value
**Demographic and Clinical Data**	Age in years	48.8 ± 8.2	49.7 ± 8.0	47.4 ± 8.2	0.261
	Gender [woman], %	67 (54)	61 (29)	78 (25)	0.095
	Hypertension, %	45 (36)	54 (26)	31 (10)	0.043
	Dyslipidemia, %	71 (57)	85 (41)	50 (16)	<0.001
	Insulin resistance, %	75 (60)	83 (40)	63 (20)	0.035
	Type 2 diabetes, %	28 (22)	33 (16)	19 (6)	0.146
	Additional MetS criteria ^#^, %				
	0	19 (15)	0 (0)	47 (15)	<0.001
	1	21 (17)	0 (0)	53 (17)	
	2	33 (26)	54 (26)	0 (0)	
	3	20 (16)	33 (16)	0 (0)	
	4	7 (6)	13 (6)	0 (0)	
	Alcohol consumption, %	7.5 (6)	6.2 (3)	9.4 (3)	0.603
	Tobacco smoking, %	19 (15)	21 (10)	15 (5)	0.559
**Anthropometric Measures**	BMI, Kg/m^2^	33.8 ± 4.2	35.1 ± 3.2	32.1 ± 3.8	0.002
	Waist circumference, cm	105.1 ± 13.0	111.3 ± 10.2	95.9 ± 11.2	<0.001
	Waist-hip ratio	0.90 ± 0.09	0.94 ± 0.09	0.84 ± 0.07	<0.001
	Fat mass, %	36.8 ± 6.1	37.6 ± 5.6	35.7 ± 6.5	0.185
**Metabolic Profile**	Glucose, mg/dL	105 ± 27	112 ± 33	95 ± 9	<0.001
	Insulin, mU/L	23.2 ± 22.9	27.8 ± 17.7	16.6 ± 10.7	0.018
	HOMA-IR	6.62 ± 9.22	8.5 ± 11.5	3.97 ± 2.78	0.016
	HbA1c, %	6.22 ± 1.64	6.65 ± 1.97	5.57 ± 0.47	0.001
	Total cholesterol, mg/dL	210 ± 39	214 ± 43	206 ± 33	0.356
	LDL cholesterol, mg/dL	128 ± 32	129 ± 35	126 ± 27	0.710
	HDL cholesterol, mg/dL	51 ± 14	49 ± 16	56 ± 13	0.028
	VLDL cholesterol, mg/dL	30 ± 15	34 ± 16	23 ± 12	0.001
	Triglycerides, mg/dL	149 ± 77	172 ± 80	116 ± 59	0.001

Number of individuals is in parenthesis. Categorical variables are shown as percentage and were compared by chi-square. Continuous variables are shown as mean ± SD and compared by *t*-test or Mann–Whitney U test. BMI: Body mass index; HOMA-IR: Homeostasis model assessment of insulin resistance; HbA1c: Glycated hemoglobin; LDL: Low-density lipoprotein; HDL: High-density lipoprotein; VLDL: Very low-density lipoprotein. (#) All patients had abdominal obesity, then they have at least 1 MetS criteria and the can meet 0 to 4 additional criteria of MetS.

**Table 2:** miRNAs differentially expressed in peripheral blood of MetS and non-MetS obese subjects (Screening study).

miRNA	Fold Change	*p*-Value	FDR
let-7a-1	−2.22433	9.00 × 10^−6^	0.000531
let-7f-1	−2.9723	0.000123	0.004344
let-7g	−2.03151	0.000827	0.020901
let-7i	−2.48811	7.25 × 10^−7^	0.000128
mir-28	−1.59773	0.002377	0.04208
mir-30d	−1.57958	0.001446	0.02843
mir-155	−1.96078	2.07 × 10^−5^	0.000916
mir-181a	−2.08434	8.60 × 10^−6^	0.000531
mir-363	−1.6846	0.000663	0.019573
mir-1839	−1.81056	0.001164	0.025752

Fold change represents the differential expression of miRs between MetS (*n* = 6) and non-MetS (*n* = 6) groups. Comparative analysis was performed by the Baggerley test corrected by False Discovery Rate (FDR) for multiple tests using the CLC Genomic Workbench software (Qiagen, Hilden, Germany).

**Table 3:** Significant correlation of miR-155 with *CEBPB* expression and other variables in obese patients (*n* = 80).

Variables	*r*	*p*-Value
*CEBPB* mRNA	−0.431	<0.001
HOMA-IR	−0.256	0.036
Adiponectin	0.292	0.017
Fibrinogen	−0.252	0.029

The correlation was evaluated using the Spearman correlation test. *r*: Correlation coefficient; HOMA-IR: Homeostasis model assessment of insulin resistance.

## Data Availability

Data are not available due to ethical restrictions.

## Supplementary Materials

The following are available online at https://www.mdpi.com/1422-0067/22/3/1468/s1.

## Abbreviations

BMI	Body mass index
CEBP	CCAAT Enhancer binding protein beta gene
FDR	False discovery rate
GLP-1	Glucagon like peptide 1
HbA1c	Glycated hemoglobin
HDL	High-density lipoprotein
HOMA-IR	Homeostasis model assessment of insulin resistance
hsCRP	High-sensitivity C-reactive protein
IL-1β	Interleukin 1β
IL-6	Interleukin 6
IL-10	Interleukin 10
IPA	Ingenuity Pathway Analysis
KRAS	KRAS proto-oncogene, GTPase
LDL	Low-density lipoprotein
MetS	Metabolic Syndrome
miRNAs	microRNAs
NF-kB	Nuclear factor Kappa B
PBMC	Peripheral blood mononuclear cells
RIN	RNA integrity number
RT-qPCR	Reverse transcription quantitative PCR
SOCS1	Suppressor of cytokine signaling 1
TNF-α	Tumor necrosis factor alpha
T2D	Type 2 diabetes
VLDL	Very low-density lipoprotein
