# Supplementary material for: Peripheral Blood miRome Identified miR-155 as Potential Biomarker of MetS and Cardiometabolic Risk in Obese Patients
Source: Int J Mol Sci. 2021 Feb 2;22(3):1468. doi: 10.3390/ijms22031468 (PMC7867145; doi:10.3390/ijms22031468)
Supplement: Supplementary file 1 [file ijms-22-01468-s001.pdf]

SUPPLEMENTARY MATERIAL

Table S1. Main characteristics of obese patients selected for analysis of miRNAs global expression in peripheral blood (Screening study) .

**Variables**	**MetS** (6)	**non-MetS** (6)	***p*-value**
Age, years	50.7 ± 4.9	50.8 ± 6.3	*0.960*
BMI, Kg/m^2	35.1 ± 1.8	35.0 ± 2.6	*0.920*
Waist circumference, cm	106.3 ± 11.4	102.3 ± 11.6	*0.571*
Waist-hip ratio	0.86 ± 0.05	0.84 ± 0.08	*0.510*
Fat mass, %	40.3 ± 2.25	40.1 ± 2.0	*0.852*
Glucose, mg/dL	115 ± 13	87 ± 8	*0.007*
Insulin, mU/L	24.0 ± 10.7	12.5 ± 6.1	*0.046*
HOMA-IR	6.45 ± 2.65	2.16 ± 0.43	*<0.001*
HbA1c, %	6.00 ± 0.41	6.00 ± 0.41	*0.017*
Total cholesterol, mg/dL	220 ± 47	179 ± 22	*0.135*
LDL cholesterol, mg/dL	114 ± 27	130 ± 36	*0.443*
HDL cholesterol, mg/dL	51 ± 6	47 ± 12	*0.489*
VLDL cholesterol, mg/dL	37 ± 17	19 ± 8	*0.076*
Triglycerides, mg/dL	186 ± 84	95 ± 39	*0.076*

Number of individuals is in parenthesis. Categorical variables are shown as percentage and were compared by chi-square. Continuous variables are shown as mean ± SD and compared by *t*-test or Mann–Whitney U test. BMI: Body mass index; LDL: Low-density lipoprotein; HDL: High-density lipoprotein; VLDL: Very low-density lipoprotein; HOMA-IR: Homeostasis model assessment of insulin resistance. (#) All subjects at MetS and non-MetS groups had abdominal obesity.

Table S2. Comparisons of top100 miRNAs expressed in peripheral blood of MetS (*n*= 6) and non-MetS (*n* = 6) obese subjects (Screening study).

N	miRNA	MetS<br>Average expression	Non-MetS<br>Average expression	Fold change	*p-value*	FDR
1	let-7i	170.00	333.33	-2.488	*7.25E-07*	**0.0001**
2	mir-181a	53.67	97.17	−2.084	*8.60E-06*	**0.0005**
3	let-7a-1	4366.17	10863.50	−2.224	*9.00E-06*	**0.0005**
4	mir-155	4871.83	9897.16	−1.960	*2.07E-05*	**0.0009**
5	let-7f-1	13.83	28.83	−2.972	*0.000123*	**0.0043**
6	mir-363	2958.17	4672.67	−1.685	*0.000663*	**0.0195**
7	let-7g	7716.17	17163.33	−2.032	*0.000827*	**0.0209**
8	mir-1839	58.83	94.00	−1.811	*0.001164*	**0.0257**
9	mir-30d	1378.67	2322.50	−1.580	*0.001446*	**0.0284**
10	mir-28	2611.33	7761.67	−1.598	*0.002377*	**0.0420**
11	mir-146a	232.83	192.16	1.212	*0.0036*	0.0573
12	mir-98	239.67	534.00	−2.228	*0.0039*	0.0573
13	mir-92a	20402.00	32961.50	−1.616	*0.0049*	0.0664
14	mir-127	14.83	6.67	2.225	*0.0070*	0.0879
15	mir-24	23.17	15.00	1.544	*0.0099*	0.0916
16	let-7d	858.00	1513.83	−1.764	*0.0113*	0.0936
17	mir-130b	47.67	105.50	−2.213	*0.0118*	0.0956
18	mir-130b	6.00	14.16	−2.361	*0.0124*	0.0966
19	mir-1304	19.33	9.83	1.966	*0.0214*	0.0972
20	mir-486	738110.00	824525.50	−1.117	*0.0210*	0.1002
21	mir-342	30.67	53.16	−1.733	*0.0212*	0.1002
22	mir-185	16.00	14.00	1.143	*0.0240*	0.1078
23	mir-222	31.33	70.66	−2.255	*0.0305*	0.1143
24	mir-3615	172.83	272.33	−1.575	*0.0343*	0.1179
25	mir-4732	9.67	19.67	−2.034	*0.0386*	0.1189
26	mir-22	1801.50	3697.50	−2.052	*0.0370*	0.1189
27	mir-320b	16.33	33.67	2.061	*0.0376*	0.1189
28	mir-151a	373.33	515.33	−1.380	*0.0408*	0.1240
29	mir-196b	21.67	45.83	−2.115	*0.0439*	0.1338
30	mir-19b	8.67	13.66	−1.577	*0.0473*	0.1360
31	mir-320a	946.00	1613.67	−1.706	*0.0459*	0.1370
32	mir-25	8643.33	14297.17	−1.654	*0.0503*	0.1386
33	mir-221	47.00	87.66	−1.865	*0.0508*	0.1386

34	mir-130a	151.17	323.33	−2.139	*0.0535*	0.1394
35	mir-425	1151.17	673.67	1.709	*0.0559*	0.1399
36	mir-151a	769.17	1387.00	−1.803	*0.0567*	0.1399
37	mir-181c	13.33	24.50	−1.838	*0.0684*	0.1403
38	mir-548h	6.33	18.16	−2.868	*0.0752*	0.1401
39	let-7j	36.33	118.50	−3.261	*0.0774*	0.1409
40	mir-15a	1175.50	2266.50	−1.928	*0.0775*	0.1650
41	mir-185	7.83	15.50	−1.979	*0.0770*	0.1654
42	mir-421	28.33	45.50	−1.606	*0.0810*	0.1695
43	mir-16	4861.17	10332.83	−2.126	*0.0798*	0.1725
44	mir-4732	127.33	227.83	−1.789	*0.0936*	0.1732
45	let-7e	29.50	70.00	−2.373	*0.0950*	0.1762
46	mir-99b	9.00	19.16	−2.129	*0.1061*	0.1774
47	mir-4433b	6.83	17.67	−2.585	*0.1041*	0.1814
48	mir-636	12.83	9.16	1.400	*0.1108*	0.1864
49	mir-18a	26.17	51.16	−1.955	*0.1123*	0.1864
50	mir-10a	49.00	79.67	−1.626	*0.1196*	0.1960
51	mir-18a	12.83	23.50	−1.831	*0.1215*	0.2010
52	mir-28	5.17	9.00	−1.742	*0.1250*	0.2033
53	mir-331	28.67	51.83	−1.808	*0.1276*	0.2033
54	mir-425	11.17	9.33	1.196	*0.1295*	0.2090
55	mir-29a	12.50	22.16	−1.773	*0.1302*	0.2095
56	mir-3107	7.17	14.33	−2.000	*0.1309*	0.2105
57	mir-501	23.17	48.50	−2.094	*0.1321*	0.2125
58	mir-1307	8.17	18.50	−2.265	*0.1336*	0.2145
59	mir-100	13.83	48.16	−3.482	*0.1302*	0.2155
60	mir-486-2	14.16	122.33	−8.635	*0.1417*	0.2165
61	let-7b	5255.17	7257.83	−1.381	*0.1369*	0.2179
62	mir-589	49.33	74.16	−1.503	*0.1398*	0.2199
63	mir-451a	4883.33	9422.33	−1.929	*0.1399*	0.2238
64	mir-584	152.33	213.83	−1.404	*0.1438*	0.2243
65	mir-21	163.00	259.67	−1.593	*0.1458*	0.2333
66	mir-23b	6.33	15.17	−2.395	*0.1473*	0.2407
67	mir-6511a	7.50	11.67	−1.556	*0.1496*	0.2449
68	mir-93	7.67	15.16	−1.978	*0.1435*	0.2449
69	mir-143	106.66	186.83	−1.752	*0.1469*	0.2614
70	mir-185	676.67	545.66	1.240	*0.1509*	0.2743

71	mir-199a	42.83	67.83	−1.584	*0.1539*	0.2843
72	mir-126	9.16	7.00	1.310	*0.1556*	0.2856
73	mir-1294	7.83	8.33	−1.064	*0.1548*	0.2856
74	mir-378a	227.67	329.33	−1.447	*0.1560*	0.2960
75	mir-340	6.00	10.83	−1.8056	*0.1568*	0.2998
76	mir-140	898.00	1252.17	−1.394	*0.1589*	0.3093
77	mir-409	7.50	25.16	−3.356	*0.1596*	0.3078
78	mir-378a	50.83	69.83	−1.374	*0.1600*	0.3183
79	mir-126	134.83	193.83	−1.438	*0.1618*	0.3199
80	mir-18a	5.67	8.83	−1.559	*0.1621*	0.3199
81	let-7c	37.00	35.33	1.047	*0.1659*	0.3257
82	mir-186	1805.67	2447.66	−1.356	*0.1671*	0.3257
83	mir-7706	29.66	40.50	−1.365	*0.1676*	0.3257
84	mir-93	9760.83	13560.67	−1.389	*0.1687*	0.3257
85	mir-21	18.50	25.83	−1.396	*0.1694*	0.3257
86	mir-361	28.67	40.16	−1.401	*0.1297*	0.3257
87	mir-191	9815.83	13826.67	−1.409	*0.1735*	0.3257
88	mir-26a	4462.50	6329.50	−1.418	*0.1343*	0.3257
89	mir-27b	228.33	324.00	−1.419	*0.1576*	0.3257
90	mir-652	155.50	221.50	−1.424	*0.1214*	0.3257
91	mir-30e	374.50	547.33	−1.462	*0.1721*	0.3257
92	mir-2110	5.50	8.16	−1.485	*0.1657*	0.3257
93	mir-17	13.67	20.83	−1.524	*0.1622*	0.3257
94	mir-5010	8.66	13.83	−1.596	*0.1246*	0.3257
95	mir-103a	1162.33	1527.17	−1.313	*0.1822*	0.3443
96	mir-92a	70091.50	94821.50	−1.352	*0.1770*	0.3443
97	mir-26b	1112.67	1638.50	−1.472	*0.1633*	0.3443
98	mir-125a	34.83	53.66	−1.540	*0.1641*	0.3443
99	mir-181b	89.83	100.67	−1.121	*0.1818*	0.3494
100	mir-107	65.33	86.66	−1.327	*0.1751*	0.3494

Average expression of miRNAs is shown as mean value of reads per million (RPM). Fold change represents the differential expression of miRs between MetS (n=6) and non-MetS (n=6) groups (Screening study). Comparative analysis was performed by the Baggerley test corrected by false discovery rate (FDR) for multiple tests using the CLC Genomic Workbench software (Qiagen, Hilden, Germany).Bold shows significant results.

Table S3. Main canonical pathways related to the selected miRNAs* and target genes .

IPA Canonical Pathway	Ranking	−log(*p*-value)	Target genes
Insulin Receptor Signaling	1	1,32E01	*KRAS, NRAS, INPP5D, GYS1, PPP1R7, PRKCI, HRAS, FOXO3*
PI3K/AKT Signaling	2	1,13E01	*KRAS, NRAS, INPP5D, GYS1, HRAS, FOXO3, IKBKE*
PI3K Signaling in B Lymphocytes	3	1,12E01	*KRAS, NRAS, INPP5D, PRKCI, HRAS, FOXO3, IKBKE*
IL-6 Signaling	6	5,17E-02	*KRAS, NRAS, CEBPB, SOCS1, HRAS, IKBKE*
JAK/Stat Signaling	11	8,44E00	*KRAS, NRAS, CEBPB, SOCS1, HRAS*

Analysis carried out using the canonical pathways tool of the Ingenuity Pathway Analysis (IPA; Qiagen, Redwood City, CA, USA). Ranking indicates position relative to the decreasing value of the p-value in the analysis. *miRNAs were differentially expressed in peripheral blood of the MetS (n = 6) compared to the non-MetS (n = 6) obese patients (Screening study).

Table S4. Inflammatory biomarkers and adipokine levels in obese patients (*n* = 80) according to HOMA-IR values .

**Variable**	**HOMA-IR** (25th percentile)			**HOMA-IR** (75th percentile)		
	**> 2.6**	**≤ 2.6**	***p*-value**	**> 7.6**	**≤ 7.6**	***p*-value**
Fibrinogen, mg/dL	368 (322-473)	353 (313-404)	*0.335*	416 (360-519)	353 (313-424)	0.009
hsCRP, mg/L	2.33(0.74-7.93)	1.16 (0.35-3.04)	*0.057*	3.36 (1.19-15.45)	1.79 (0.42-5.19)	0.038
IL-1β, pg/dL	0.48 (0.25-0.64)	0.18 (0.08-0.60)	*0.132*	0.51 (0.34-2.60)	0.28 (0.12-0.53)	0.158
Adiponectin, μg/mL	20.8 (7.0-50.3)	29.5 (13.4-120.3)	*0.309*	17.2 (9.2-25.6)	28.4 (14.9-86.4)	0.048
Leptin, ng/mL	24.3 (16.6-38.2)	23.7 (11.9-31.1)	*0.488*	25.2 (19.5-36.5)	22.5 (10.6-37.6)	0.432
Resistin, ng/mL	42.8 (35.6-52.7)	46.5 (34.4-66.3)	*0.748*	45.6 (36.3-57.0)	40.3 (30.7-51.8)	0.373

Values are shown as median (95% confidence interval) and were compared by Mann–Whitney test. Patients were grouped according to HOMA-IR cut-off values. HOMA-IR >2.6: 25th percentile; HOMA-IR >7.6: 75th percentile. Coincidently, HOMA-IR 25th percentile (2.6) correspond to the clinical criteria for insulin resistance.

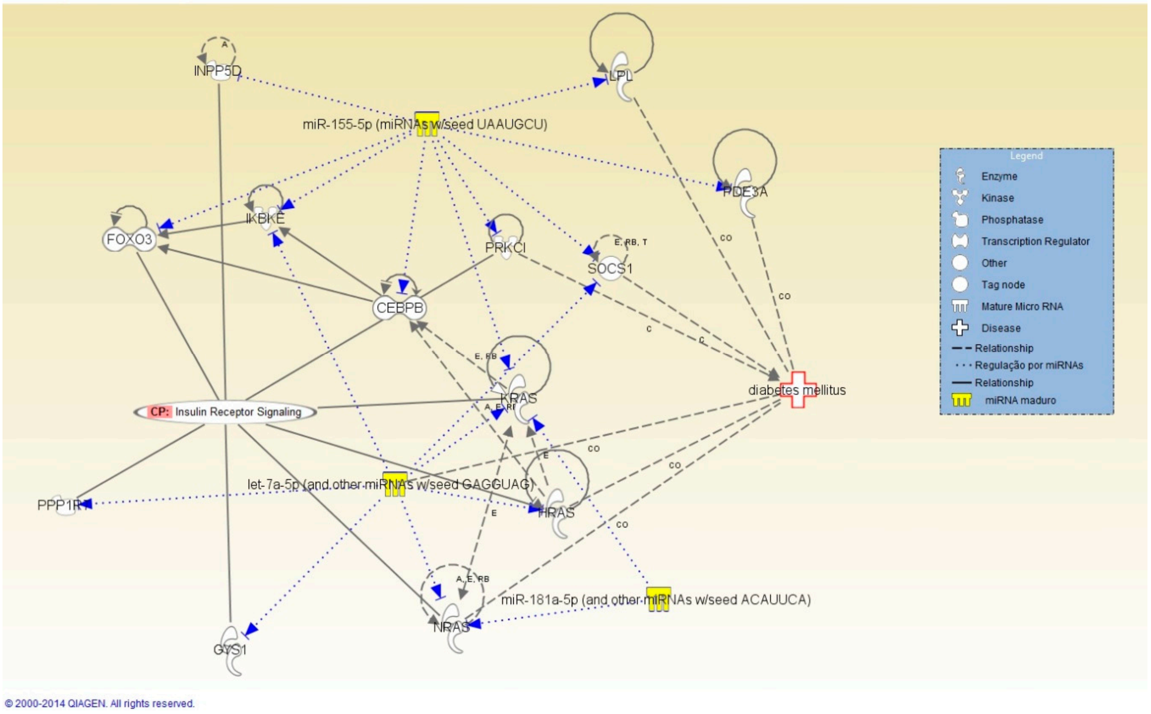

Figure S1. Interactions of the selected miRNAs (mir-155, miR-181 and let-7a) and target genes involved in the regulation of the insulin receptor signaling pathway. The target genes were selected using the miRNA Target Filter tool and the figure was constructed using the Path Designer tool of the Ingenuity Pathway Analysis (IPA; Qiagen, Redwood City, CA, USA).

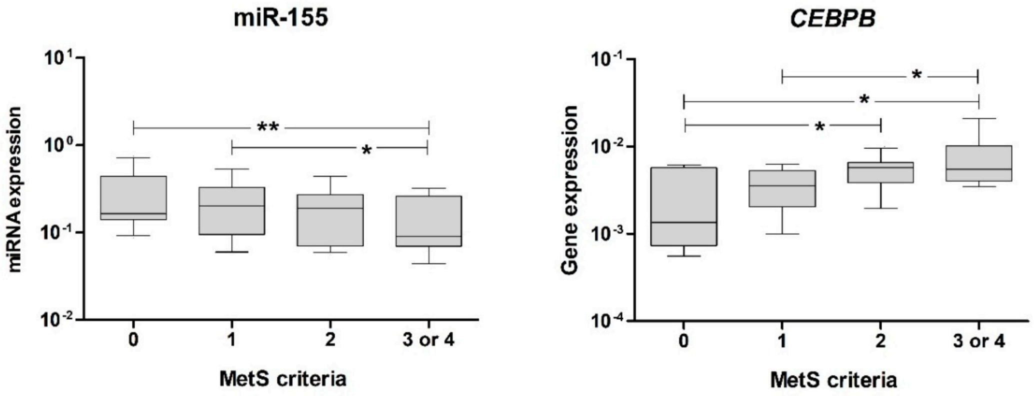

Figure S2. Expression of miR-155 and *CEBPB* mRNA according to the number of additional MetS diagnostic criteria in obese patients (*n* = 80). Data are shown as box plots and were compared by Kruskal–Wallis test and Dunn's post-hoc test. As all patients had central obesity, and additional risk factors were considered as the presence of any other criteria for MetS diagnosis according to IDF. Patients with three or four additional criteria were grouped together due to the small number of subjects in the last group. (*), $p < 0.05$; (**), $p < 0.01$.
